# High-efficiency isolation of fetal nucleated red blood cells for non-invasive prenatal diagnosis *via* a cascaded microfluidic platform

**DOI:** 10.1039/d6ra02995g

**Published:** 2026-07-03

**Authors:** Hongtao Feng, Yuqing Huang, Weiliang Shu, Fengshan Shen, Bin Huang, Jiaxin Zhang, Shunchan Gao, Hui Liang, Likuan Xiong, Kaidong Ma, Zongbin Liu, Yan Chen

**Affiliations:** a Shenzhen Institutes of Advanced Technology, Chinese Academy of Sciences Shenzhen 518055 China yan.chen@siat.ac.cn ht.feng@siat.ac.cn; b Shenzhen Zigzag Biotechnology Co., Ltd Shenzhen 518107 China Zongbin.liu@zigbio.com; c Shenzhen Baoan Women's and Children's Hospital Shenzhen 518102 China; d Center of Obstetrics and Gynecology, Peking University Shenzhen Hospital Shenzhen 518036 China; e Shenzhen Raymind Biotechnology Co., Ltd Shenzhen 518129 China

## Abstract

Non-invasive prenatal diagnosis (NIPD) utilizing fetal nucleated red blood cells (fNRBCs) holds great promise for comprehensive genetic analysis; however, its clinical application is challenged by the extreme rarity of these cells in maternal peripheral blood. Herein, we present a two-step cascaded microfluidic platform designed for the high-efficiency isolation of fNRBCs. This integrated system employs a deterministic lateral displacement (DLD) microfluidic chip for the initial label-free enrichment of large cells, including fNRBCs, based on size. The enriched product is subsequently subjected to negative purification using a magnetic microfluidic chip, where residual white blood cells are depleted following incubation with CD45-coated magnetic beads. Using spiked K562 cells as a model, the device demonstrated a consistently high recovery efficiency exceeding 90%. In a clinical validation with 20 maternal blood samples, fNRBCs (identified as DAPI^+^/CD71^+^/CD45^−^) were successfully isolated from all subjects, with yields ranging from 10 to 83 cells per milliliter. Furthermore, the fetal origin of the isolated cells was definitively confirmed *via* fluorescence *in situ* hybridization (FISH) using Y-chromosome-specific probes in samples from pregnancies with male fetuses. These results demonstrate that our cascaded microfluidic platform provides a highly efficient, practical, and reliable approach for fNRBC isolation, highlighting its significant potential for cell-based non-invasive prenatal testing.

## Abstract 2

A cascaded platform combining label-free DLD enrichment and immunomagnetic negative purification achieves high-efficiency fNRBC isolation for non-invasive prenatal diagnosis.

## Graphical abstract

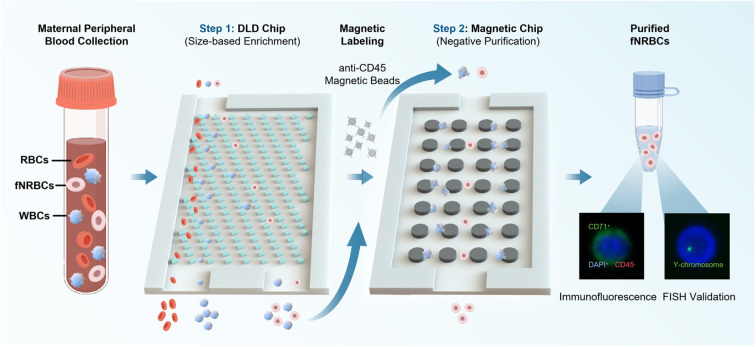

## Introduction

Prenatal diagnosis is a critical approach for the early detection of genetic disorders, playing a vital role in clinical management and the prevention of birth defects.^1,2^ While traditional invasive procedures—such as amniocentesis and chorionic villus sampling (CVS)—remain the gold standard for acquiring fetal genetic material, they are associated with inherent risks, including infection and miscarriage.^3–5^ To address these safety concerns, non-invasive prenatal testing (NIPT) has been developed as a reliable alternative.^6^ NIPT primarily targets two sources of fetal genetic material in maternal peripheral blood: cell-free fetal DNA (cffDNA) and circulating fetal cells. Currently, cffDNA-based screening is extensively utilized for detecting common aneuploidies, demonstrating high sensitivity (≥99% for trisomy 21) and specificity.^7^ However, the broader application of cffDNA for detecting sub-chromosomal abnormalities, such as micro-deletions and duplications, remains challenging. This limitation is primarily due to the fragmented nature of cffDNA and the high interference from background maternal DNA.^8^

In contrast to cffDNA, circulating fetal nucleated cells possess intact nuclei containing the complete fetal genome, thereby enabling comprehensive genetic analysis beyond simple aneuploidy detection.^9^ Four primary types of fetal cells have been identified in maternal peripheral blood: trophoblasts, lymphocytes, hematopoietic stem cells, and fetal nucleated red blood cells (fNRBCs).^10^ Among these candidates, fNRBCs demonstrate unique advantages for NIPD applications, particularly in early gestation. Their suitability is driven by two distinctive characteristics: (1) distinct morphological features combined with a complete genetic complement allow for precise differentiation from maternal blood cells;^11^ and (2) the expression of specific surface antigens—such as the transferrin receptor (CD71), thrombospondin receptor (CD36), and glycophorin A (GPA)—provides robust targets for specific cellular identification and isolation.^12,13^

However, the clinical utility of fNRBCs is severely hampered by their extreme scarcity in maternal peripheral blood, where they exist alongside a vast background of maternal cells.^14^ This high rarity poses a significant technical challenge: isolating target cells with both high efficiency and purity is difficult but essential for reliable downstream analysis. To address this, various enrichment strategies have been developed, including density gradient centrifugation, magnetic-activated cell sorting (MACS),^15,16^ fluorescence-activated cell sorting (FACS),^17^ and combinatorial approaches. Typically, these techniques fall into two categories: (1) label-free approaches, which exploit intrinsic physical properties such as cell size, deformability, and density; and (2) immunoaffinity-based methods, which rely on antigen–antibody interactions for targeted capture. While immunoaffinity methods (utilizing antibodies immobilized on beads, substrates, or filters) offer high specificity, they face inherent limitations: the strong binding required for capture often complicates cell release, potentially compromising cell viability and interfering with subsequent molecular analyses.

In contrast, label-free microfluidic approaches have gained prominence due to their distinct advantages, including high efficiency, minimal cell damage, and cost-effectiveness. Among these strategies, Deterministic Lateral Displacement (DLD) stands out for its ability to precisely sort cells based on critical size and deformability. In previous studies, our group successfully developed a DLD-based platform for the isolation of Circulating Tumor Cells (CTCs), achieving high sensitivity, specificity, and—crucially—high cell viability suitable for downstream analysis.^18,19^ Notably, fNRBCs share a key physical attribute with CTCs: they are significantly larger than the majority of white blood cells (WBCs) and red blood cells (RBCs).^20^ Leveraging this morphological similarity, and inspired by our success with CTCs, we adapted and optimized our established DLD architecture to specifically target the efficient capture of fNRBCs from maternal blood.

In this work, we present a two-step cascaded microfluidic platform designed for the high-efficiency isolation of fNRBCs from maternal peripheral blood. This integrated system synergizes two distinct technologies: an initial deterministic lateral displacement (DLD) module for high-throughput, size-based enrichment, followed by a magnetic separation module for negative purification. The DLD chip is engineered to process blood samples with minimal pre-processing, effectively reducing the background of red blood cells. To maximize purity, the DLD-enriched product is subsequently incubated with CD45-conjugated magnetic beads, allowing for the specific depletion of residual maternal white blood cells (WBCs) in the magnetic chip. We quantitatively evaluated the platform's performance using K562 cell spike-in experiments and validated its clinical utility in a cohort of 20 pregnant women (including 4 with confirmed male fetuses) alongside 5 non-pregnant controls. Isolated cells were characterized *via* immunofluorescence staining, and crucially, their fetal origin was definitively confirmed using fluorescence *in situ* hybridization (FISH) with Y-chromosome-specific probes. We believe this integrated microfluidic device represents a significant advancement in fNRBC isolation, holding great promise for facilitating reliable, cell-based non-invasive prenatal diagnostics.

## Experimental

### Reagents and materials

Polydimethylsiloxane (PDMS; Sylgard 184, Dow Corning) was purchased from Chip Technology Co., Ltd. CD45 magnetic beads and antibodies (anti-CD71-FITC and anti-CD45-PE) were obtained from STEMCELL Technologies and Miltenyi Biotec, respectively. Bovine serum albumin (BSA), 4′,6-diamidino-2-phenylindole dihydrochloride (DAPI), and other general laboratory reagents were sourced from Sigma-Aldrich. The green Centromeric Enumeration Probe (CEP) for the human Y chromosome was acquired from GeneCopoeia Inc.

### Fabrication of microfluidic chips

The DLD chip was manufactured using standard photolithography and soft lithography techniques. Briefly, a master mold was created by patterning SU-8 3050 photoresist on a silicon wafer using an EVG mask aligner. A PDMS prepolymer mixture (base-to-curing agent ratio of 10 : 1, w/w) was poured onto the master mold and degassed under vacuum for 20 minutes to eliminate air bubbles. The PDMS was subsequently cured at 80 °C for 30 minutes. Following curing, the solidified PDMS slab was peeled from the mold, and inlet/outlet ports were punched using a biopsy punch. Finally, the patterned PDMS layer and a glass slide were activated *via* oxygen plasma treatment and permanently bonded to form the sealed microfluidic device.

For the magnetic chip, an SU-8 mold with a height of 180 µm was employed. This mold featured an array of micro-cavities (300 µm diameter, 700 µm pitch) which were manually packed with micron-sized iron powder (Fe powder). The PDMS prepolymer mixture was then poured over the powder-filled mold. During vacuum degassing, the PDMS infiltrated the interstices between the iron particles, resulting in the formation of high-concentration iron-PDMS composite micro-pillars upon curing. After peeling and punching, the device was bonded to a 0.17 mm-thick glass coverslip *via* oxygen plasma treatment. To generate the requisite magnetic gradient, permanent magnets were positioned directly beneath the assembled device.

### COMSOL simulation

Numerical simulations were performed using COMSOL Multiphysics to characterize the physical fields within the magnetic chip. Two separate steady-state studies were conducted. First, the static magnetic flux density distribution generated by the permanent neodymium (NdFeB) magnets was calculated using the Magnetic Fields physics interface. Independently, the fluid flow velocity field was resolved using the Laminar Flow interface, imposing an inlet velocity boundary condition of 1 × 10^−3^ m s^−1^. The results from these simulations were analyzed to quantify the magnetic environment and hydrodynamic profile within the microchannel, providing the basis for subsequent force analysis.

### Cell culture and model cell preparation

The human chronic myelogenous leukemia cell line, K562, was obtained from the American Type Culture Collection (ATCC). Cells were cultured in Roswell Park Memorial Institute (RPMI) 1640 medium supplemented with 10% (v/v) fetal bovine serum (FBS) and 1% (v/v) penicillin-streptomycin (all from Gibco, USA). The cultures were maintained at 37 °C in a humidified atmosphere containing 5% CO_2_. In this study, K562 cells were selected as a surrogate model for fNRBCs due to their comparable diameter and the expression of characteristic erythroid surface markers, which align with the phenotypic and morphological profiles of primary fNRBCs.^4,21^ Crucially, the CD45-negative nature of K562 cells allows for the rigorous validation of our leukocyte depletion modules without the risk of non-specific capture associated with leukocyte-specific markers. Prior to microfluidic experiments, K562 cells were harvested, quantified, and pre-stained with Vybrant® DyeCycle™ Green stain (Invitrogen) to facilitate subsequent identification and recovery analysis.

### Cell recovery assay

K562 cells were spiked into diluted blood samples obtained from healthy donors at predetermined concentrations. The spiked sample was then infused into the DLD chip through the blood inlet at a flow rate of 0.5 mL min^−1^, while a buffer solution was introduced *via* the adjacent inlet at 0.2 mL min^−1^. The target K562 cells, along with some larger leukocytes, were collected from the designated fNRBCs outlet.

The collected cell suspension was subsequently incubated with CD45 antibody-conjugated magnetic beads for 20 minutes at 37 °C to label unwanted leukocytes. Following incubation, the mixture was processed through the magnetic purification chip to deplete these CD45-positive cells. Finally, the pre-stained K562 cells collected from the chip were counted under a fluorescence microscope to determine the recovery efficiency. Each experiment was performed in triplicate. The recovery efficiency (η) was calculated as follows:
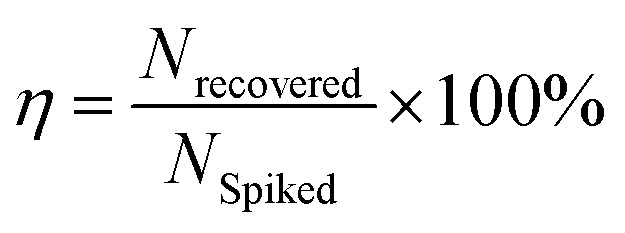
where *N*_recovered_ represents the count of target cells collected from the chip, and *N*_Spiked_ denotes the initial number of cells added to the sample.

### Blood sample collection and processing

Peripheral blood samples (gestational age: 14–37 weeks) and human fetal umbilical cord blood samples (gestational age: 38–40 weeks) were collected from healthy donors, parturient women, and pregnant volunteers following a protocol approved by the Institutional Review Board (IRB approval no.: SIAT-IRB-190315-H0337). All participants provided written informed consent prior to enrolment in this study. The cord blood was obtained immediately after delivery, and all whole blood specimens were collected in anticoagulant tubes containing EDTA-K2 and processed within 8 hours of collection to ensure cell viability. For microfluidic processing, whole blood samples were diluted with phosphate buffered saline (PBS) (*e.g.*, 3 mL PBS for 5 mL blood) to reduce sample viscosity, thereby facilitating stable perfusion through the cascaded DLD and magnetic separation modules.

### Cell viability assay

Cell viability was analyzed using a live/dead dual-staining assay. The recovered cells were incubated with an acridine orange (AO) and propidium iodide (PI) working solution (5 µg mL^−1^ AO and 5 µg mL^−1^ PI in PBS) for 10 min at room temperature in the dark. Viable cells with intact membranes display yellow-green fluorescence (AO^+^/PI^−^), whereas non-viable cells with compromised membranes exhibit red fluorescence (AO^+^/PI^+^). The cells were imaged using a fluorescence microscope. Cell viability was calculated using the following equation:
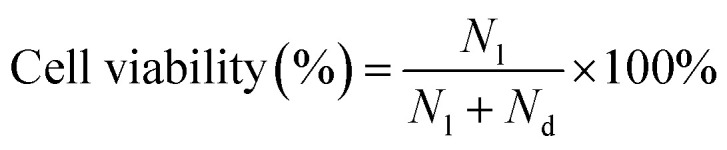
where *N*_l_ represents the number of live cells, and *N*_d_ is the number of dead cells.

### Immunofluorescence

Cells isolated by the microfluidic platform were seeded into 96-well plates pre-coated with poly-l-lysine (Sigma-Aldrich) to promote adhesion. The cells were fixed with 4% paraformaldehyde (PFA) for 15 minutes at room temperature, followed by three washes with phosphate-buffered saline (PBS). Permeabilization conditions were optimized using a mild concentration of Triton X-100 (0.04% for 10 min) to facilitate the nuclear entry of the DAPI counterstain while effectively preserving cell membrane integrity, thereby preventing the loss of critical CD45 and CD71 surface antigens. To minimize non-specific binding, samples were blocked with 3% bovine serum albumin (BSA) for 1 hour at room temperature. Subsequently, the cells were incubated overnight at 4 °C with an antibody cocktail containing anti-CD45-PE (1 : 100 dilution) and anti-CD71-FITC (1 : 100 dilution). After washing with PBS, nuclei were counterstained with 10 µg mL^−1^ 4′,6-diamidino-2-phenylindole (DAPI) for 10 minutes in the dark. Finally, the samples were washed thoroughly with PBS and imaged using a fluorescence microscope.

### Fluorescence *in situ* hybridization (FISH) analysis

To verify the fetal origin of isolated cells, FISH analysis was performed using a Y-chromosome-specific probe. Initially, cells were permeabilized in a hypotonic solution (0.4% KCl and 0.8% sodium citrate) for 20 minutes at 37 °C. Fixation was subsequently carried out using freshly prepared Carnoy's fixative (ethanol:acetic acid, 3 : 1 v/v) for 8 minutes at room temperature. Following centrifugation, 5–10 µL of the cell suspension was spotted onto poly-l-lysine-coated slides and baked at 56 °C for 30 minutes to ensure immobilization. The slides were pretreated with 0.2% NP-40 in 2 × SSC buffer at 37 °C for 10 minutes, followed by dehydration through a graded ethanol series (70%, 90%, and 100%; 1 minute each) and air-drying. A Centromeric Enumeration Probe (CEP) mix specific for the Y chromosome was applied to the target area. Co-denaturation of chromosomal DNA and probes was performed at 75 °C for 15 minutes, followed by overnight hybridization at 42 °C in a humidified chamber. Post-hybridization, unbound probes were removed *via* stringent washing: first in 0.1% NP-40/0.5 × SSC at 73 °C for 5 minutes, and then in the same solution at room temperature for 1 minute. After a brief dehydration in 70% ethanol, nuclei were counterstained with 15 µL of DAPI (10 µg mL^−1^). Finally, slides were coverslipped, incubated in the dark for 15 minutes, and visualized using a fluorescence microscope.

## Results and discussion

### Working principle of the integrated microfluidic platform

**Fig. 1:**
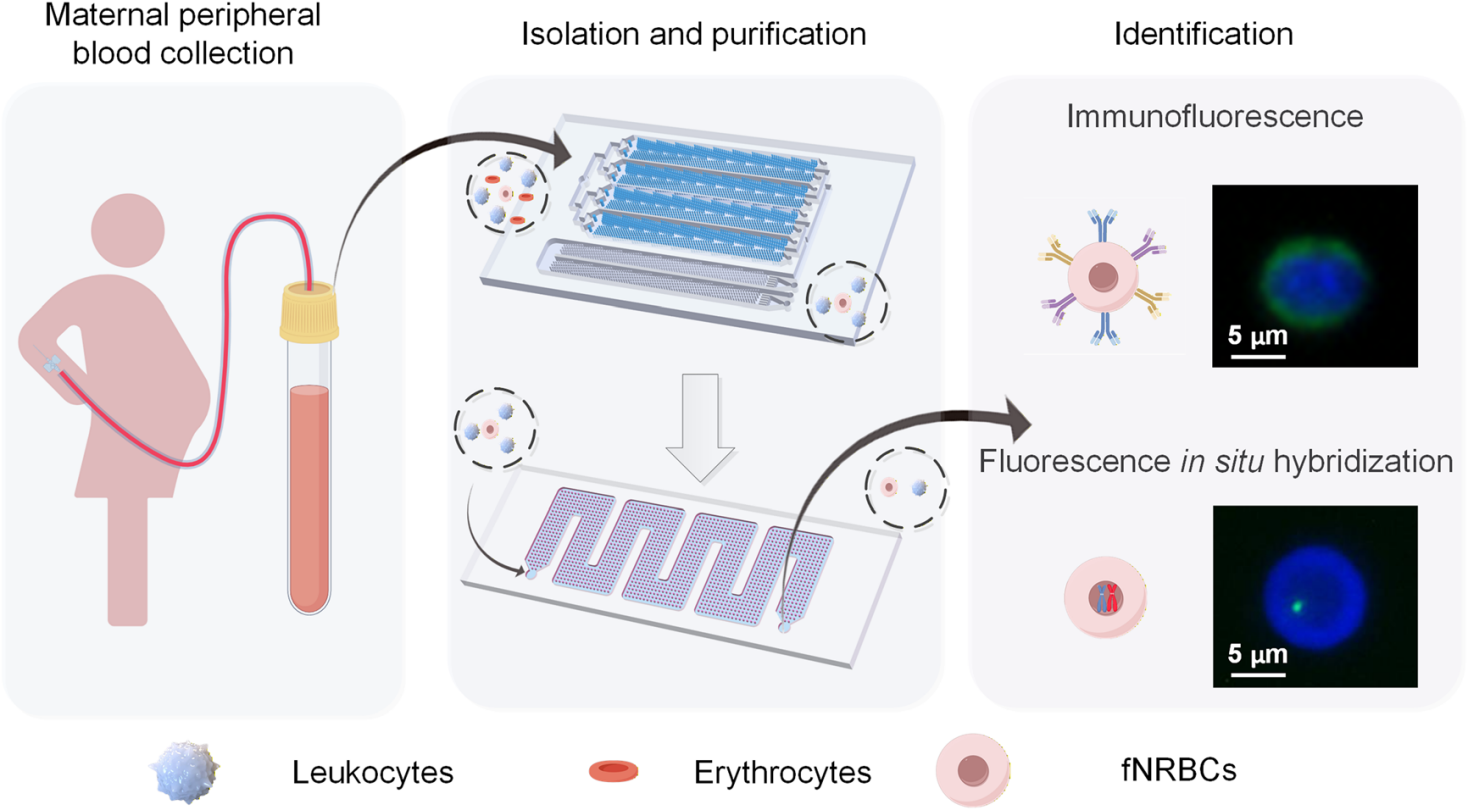
Schematic workflow of the integrated microfluidic platform for the isolation and identification of fNRBCs from maternal peripheral blood.

To address the challenge of isolating extremely rare fNRBCs from the complex background of maternal blood, we developed an integrated microfluidic platform employing a two-step cascaded strategy: label-free size-based enrichment followed by immunomagnetic negative purification (Fig. 1). Step 1: size-based Enrichment. The initial enrichment utilizes a Deterministic Lateral Displacement (DLD) microfluidic chip. The micropillar arrays are engineered with a critical diameter (*D*_c_) of approximately 8 µm to discriminate cells based on size. Consequently, larger targets like fNRBCs (∼10 µm) are laterally displaced into the “bumping” mode toward the collection outlet, while smaller maternal components—such as erythrocytes (∼5–7 µm) and lymphocytes (∼6–9 µm)—follow the “zigzag” mode to the waste outlet. To ensure separation stability and minimize shear-induced damage to fragile fetal cells, the chip incorporates a co-flow buffer sheath and a two-stage sorting architecture. Step 2: negative purification. The second stage targets the removal of residual leukocytes (WBCs) that may co-elute due to size overlap. The DLD-enriched fraction is incubated with anti-CD45-conjugated magnetic beads, followed by passage through a magnetic microfluidic module to specifically deplete CD45^+^ cells. This synergistic workflow effectively minimizes background noise while preserving fNRBC viability, thereby ensuring high purity for reliable downstream genetic analysis.

The performance of the integrated platform was rigorously validated through a dual-identification strategy: phenotypic characterization *via* immunofluorescence staining and definitive genotypic verification of fetal origin using fluorescence *in situ* hybridization (FISH). This hybrid architecture offers distinct advantages over traditional methods. By leveraging label-free size enrichment, the system avoids the cellular stress and potential damage often associated with antibody-mediated positive capture. Simultaneously, the targeted immunomagnetic depletion of residual leukocytes significantly enhances purity without compromising the integrity of the fNRBCs. Consequently, this workflow ensures the high-throughput, reproducible isolation of viable fetal cells, minimizing contamination risks and facilitating reliable cell-based non-invasive prenatal diagnosis.

### High-efficiency enrichment *via* the DLD microfluidic chip

**Fig. 2:**
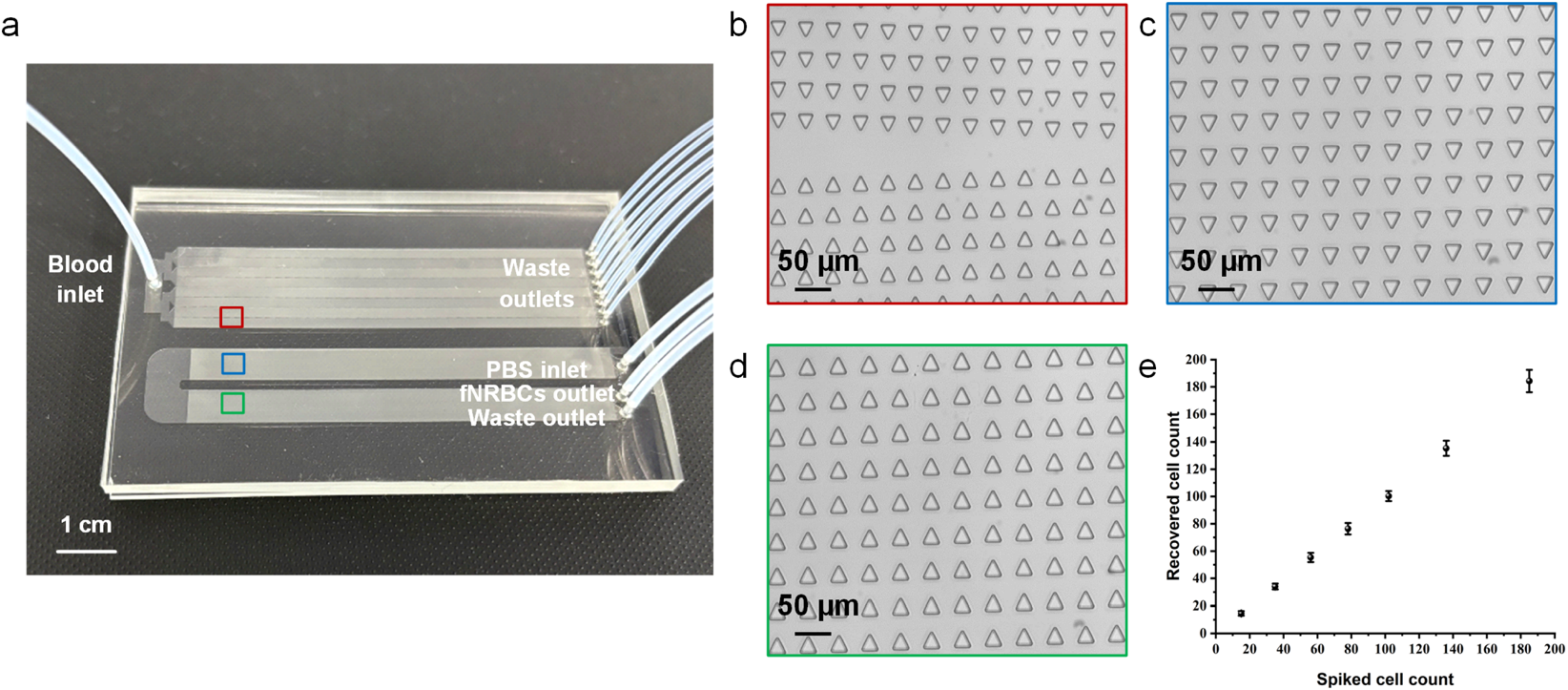
Design and validation of the DLD microfluidic chip. (a) Photograph of the fabricated device showing the fluidic inlets and outlets. (b–d) Micrographs of the triangular micropillar arrays corresponding to the red (b), blue (c), and green (d) frames in (a). (e) Quantitative analysis showing the linear correlation between spiked and recovered K562 cells.

Over recent years, our team has continuously optimized DLD architectures for precise, size-based cell separation. The integrated DLD microfluidic device fabricated for this study is illustrated in Fig. 2a. The chip (76 mm × 50 mm) is composed of two cascaded functional modules: a bulk blood cell depletion module and a refined fNRBC separation module. The core functionality of these modules relies on the deterministic lateral displacement effect induced by periodic arrays of triangular micropillars, whose microscopic structures are shown in Fig. 2b–d. The fundamental principle is that as blood flows through the array, particles smaller than *D*_c_—such as RBCs—follow the fluid streamlines in a “zigzag” mode, while particles larger than *D*_c_—such as fNRBCs and CTCs—are deterministically deflected in a “bumping” mode. Quantitatively, *D*_c_ is determined by the established empirical formula: *D*_c_ = 1.4 *G ε*^0.48^, where *G* is the lateral gap distance and *ε* is the row shift fraction (*ε* = tan *θ*).^22^ For our specific triangular micropillar design (pillar width = 20 µm, *G* = 30 µm, *θ* = 2°), the theoretical *D*_c_ is calculated to be ∼8 µm. Building upon our previous optimization of DLD architectures for rare cell isolation, this threshold optimally ensures that target fNRBCs are laterally deflected, while the vast majority of mature erythrocytes pass through along the streamlines. The precise tuning of these parameters is critical: a smaller *D*_c_ would cause severe erythrocyte contamination in the collected fraction, whereas a larger *D*_c_ would erroneously direct rare fNRBCs into the waste, reducing recovery. Furthermore, the 30 µm gap ensures completely clog-free operation for the target cells. Detailed schematics of these structural parameters are provided in Fig. S1.

To maximize performance based on this design, we implemented a cascaded two-stage sorting strategy: Stage 1 (Bulk Depletion): The sample enters *via* a single inlet. The primary array removes the majority of RBCs, significantly reducing sample complexity and cell–cell interactions. Stage 2 (Refined Separation): The pre-processed sample then flows into the second unit. Although its structural parameters are identical to the first stage, the prior removal of the massive RBC background drastically reduces cell crowding and steric hindrance. While extreme cell concentrations in the first stage can disrupt local laminar flows and cause collision-induced mis-sorting, Stage 2 operates in a substantially cleaner suspension. This allows the remaining nucleated cells to strictly follow their theoretical hydrodynamic trajectories without physical interference, yielding refined separation and enhanced resolution.

To quantify recovery efficiency, K562 cells were selected as a model for fNRBCs due to their comparable diameter (∼10 µm) and morphology, consistent with reported fNRBC characteristics. K562 cells pre-stained with Vybrant® DyeCycle™ Green were spiked into healthy blood samples. As shown in Fig. 2e, the DLD chip demonstrated a recovery efficiency exceeding 97%. However, despite efficient RBC removal, the residual leukocyte count remained sufficient to interfere with downstream analysis, necessitating a secondary purification step.

### Design and simulation of the magnetic depletion module

**Fig. 3:**
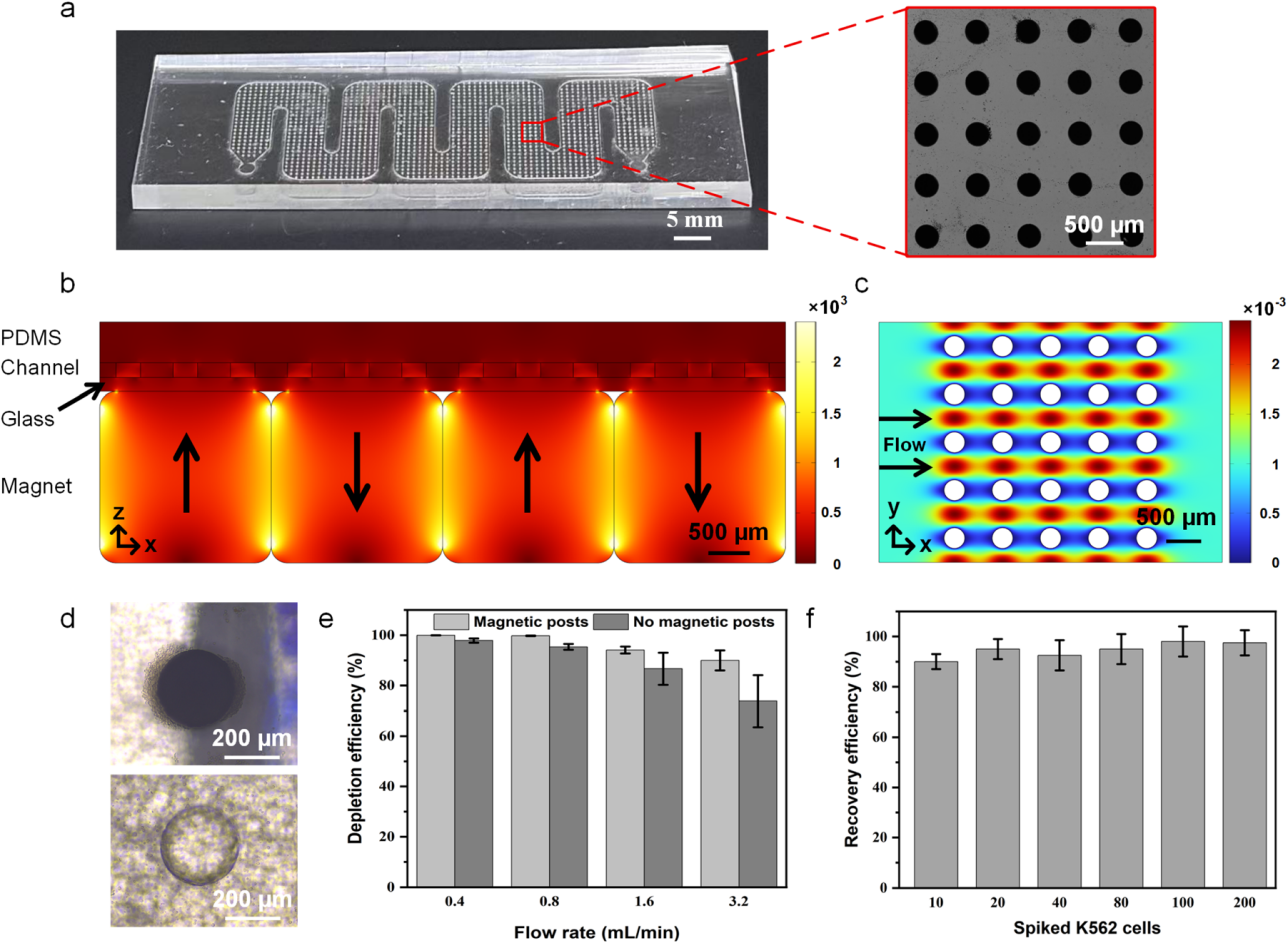
Design and characterization of the magnetic depletion module. (a) Photograph of the assembled PDMS-glass microfluidic chip with serpentine channels, and enlarged view of the magnetic pillar array (red frame). (b) Magnetic field simulation showing the influence of the iron pillar array in the PDMS chip (unit: mT). (c) Simulation of flow velocity field (inflow velocity = 1 × 10^−3^ m s^−1^) in the magnetic microfluidic chip (unit: m s^−1^). (d) Comparison of leukocyte capture on Fe-doped (top) *versus* non-doped (bottom) micropillars. (e) Depletion efficiency of WBCs at flow rates of 0.4–3.2 mL min^−1^. (f) Overall recovery efficiency of the integrated system for spiked K562 cells (simulated fNRBCs).

To eliminate residual leukocytes, we developed an integrated magnetic microfluidic chip for negative purification (Fig. 3a). Following DLD enrichment, the cell suspension is incubated with anti-CD45-conjugated magnetic beads (37 °C, 20 min) to label leukocytes. The sample is then perfused through the magnetic chip, which features a PDMS matrix embedded with high-concentration micro-iron powder, fabricated *via* soft lithography (Fig. 3a, inset).

Unlike conventional tube-based separation, this chip utilizes an array of Fe-doped PDMS micro-pillars to modulate the external magnetic field, creating a high density of strong capture sites. COMSOL simulations (Fig. 3b and S2) revealed that these Fe-doped pillars significantly amplify the local magnetic field compared to non-doped PDMS micropillars, thereby substantially enhancing the magnetic capture force exerted on the beads. Furthermore, the cumulative binding of multiple magnetic beads to a single target cell dramatically amplifies the total retention force.

To further elucidate this capture mechanism at the single-cell level, a quantitative force balance analysis was performed (detailed calculations are provided in SI Note 1). Taking a reference velocity of 1 mm s^−1^ as a conservative model based on our fluid dynamics simulations (Fig. 3c), the calculated hydrodynamic drag force acting on a leukocyte is approximately 113.1 pN. In contrast, for the 200 nm magnetic nanoparticles utilized in our assay (EasySep™ Depletion Kit), the individual magnetic retention force generated by the Fe-doped pillars is estimated at 2.89 pN. Given that a typical leukocyte possesses a vast surface area with a high density of CD45 binding sites, the cell is capable of accommodating thousands of magnetic nanoparticles (*F*_mag_ = *n*·*F*_mag_single_), ensuring the cumulative magnetic force easily overcomes the hydrodynamic drag (*F*_mag_ ≫ *F*_drag_). This substantial multivalent safety margin ensures robust capture performance even under elevated high-throughput flow rates. Furthermore, the non-uniform flow field around the micro-pillars creates localized low-velocity zones, which systematically reduce the actual drag force encountered by the cells, providing an optimal hydrodynamic environment for stable immobilization.

Experimental validation provided robust confirmation of these theoretical predictions. Microscopic inspection of the capture process (Fig. 3d) revealed a striking contrast: in the presence of Fe-doped micropillars, a dense accumulation of magnetically labeled leukocytes was observed surrounding the pillars, confirming the generation of strong localized magnetic gradients. In comparison, control structures utilizing non-doped PDMS pillars exhibited negligible cell capture under identical external magnetic fields, highlighting the critical role of the iron matrix in enhancing capture capability.

Quantitative analysis of leukocyte depletion efficiency (Fig. 3e) further substantiated the superiority of the magnetic-pillar design. At lower flow rates (0.4–0.8 mL min^−1^), the chip consistently achieved a high depletion efficiency exceeding 99%. Crucially, the iron-pillar architecture demonstrated superior stability against increasing hydrodynamic drag. When the flow rate was elevated to 1.6–3.2 mL min^−1^, the depletion efficiency of the non-doped control group declined significantly due to drag forces overcoming the magnetic retention. In contrast, our Fe-doped magnetic-pillar chip maintained high performance (retaining ∼90% efficiency even at 3.2 mL min^−1^), effectively counteracting the elevated drag force. These results confirm that the amplified magnetic gradients generated by the iron pillars are essential for ensuring high-throughput, high-purity cell isolation.

### Overall system efficiency and comparative analysis

The integrated platform demonstrated robust performance in spike-and-recovery experiments. As illustrated in Fig. 3f, the overall recovery efficiency of the combined DLD and magnetic system consistently exceeded 90% across all tested spiking levels (10–200 cells).

Notably, this performance represents a significant improvement over existing technologies. For instance, the nanostructure-based chip reported by Wei *et al.*^23^ achieved a capture efficiency of 80% with a subsequent release efficiency of 89%, resulting in a calculated net recovery of approximately 71%. In contrast, our platform maintains high efficiency (>90%) by avoiding the trade-offs associated with cell capture and release. This superior recovery rate is a critical advantage for NIPD, where the extreme rarity of fNRBCs demands minimal cell loss to ensure reliable downstream genetic analysis.

To evaluate cell integrity post-processing, the viability of the recovered target cells was assessed *via* an AO/PI dual-staining assay. The isolated cells exhibited a high viability of 95.53 ± 1.78% (representative fluorescence images are provided in Fig. S3), confirming that the cascaded microfluidic process exerts minimal stress on the fragile target cells.

### Identification and quantification of fNRBCs in clinical samples

To validate the clinical utility of the platform, we analyzed peripheral blood samples (ranging from 2.0 to 5.0 mL, as detailed in Table S1) from a cohort of 20 pregnant volunteers (gestational age: 14–37 weeks) and 5 non-pregnant controls (demographic details in Table S1). All samples were processed using the integrated microfluidic system, followed by three-color immunofluorescence characterization.

We employed a specific staining panel targeting CD71 and CD45. The transferrin receptor (CD71) is highly expressed on early erythroid progenitors (including fNRBCs) but is absent or minimally expressed on mature erythrocytes and leukocytes.^23,24^ Consequently, fNRBCs were identified as DAPI^+^/CD71^+^/CD45^−^, clearly distinguishing them from maternal leukocytes (DAPI^+^/CD45^+^/CD71^−^). Notably, unlike previous studies that utilized CD71 antibodies for positive cell capture,^4,25^ our label-free isolation strategy employs CD71 exclusively for downstream phenotypic identification, thereby preserving the native surface properties of the captured cells.

**Fig. 4:**
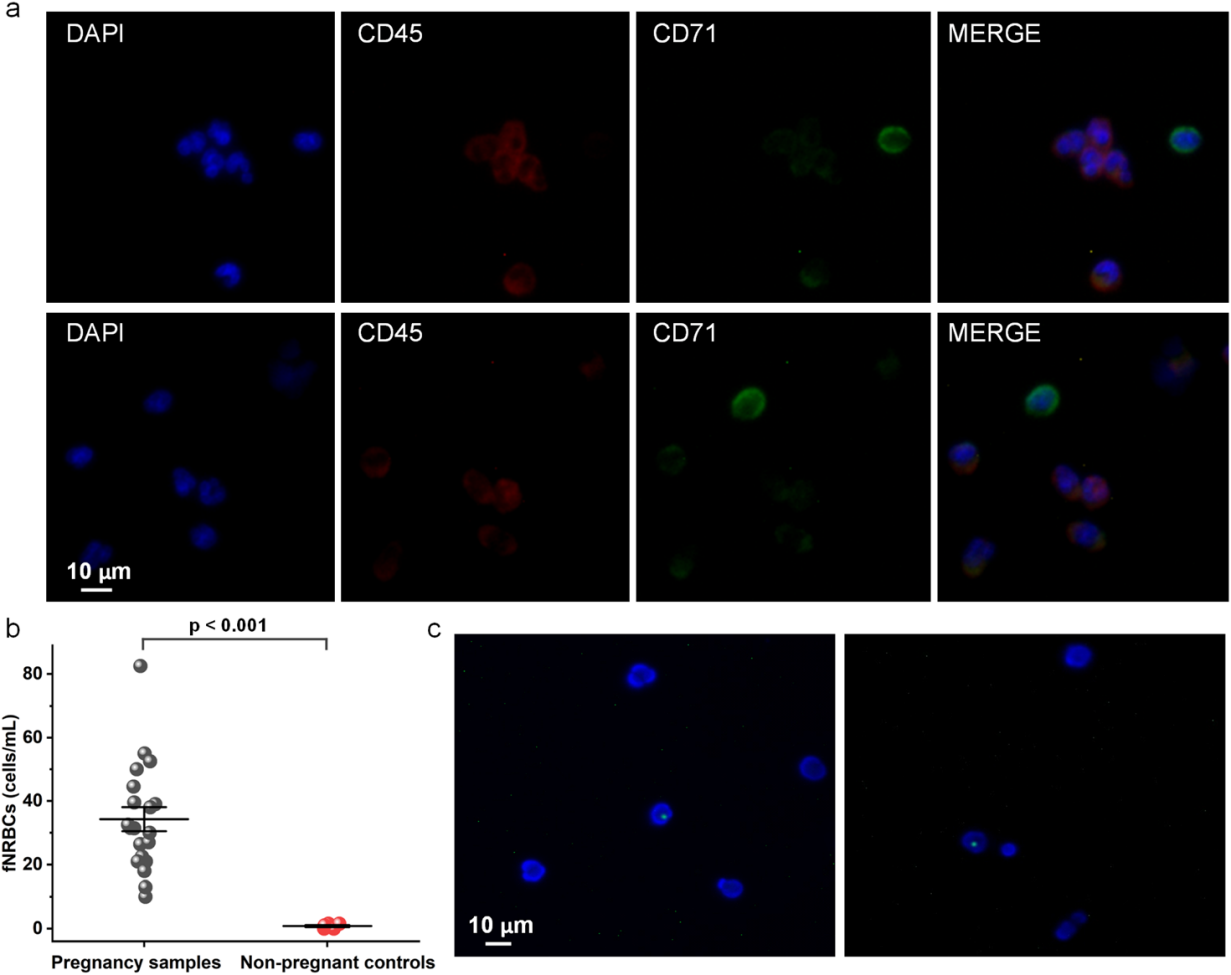
Identification and clinical validation of isolated fNRBCs. (a) Representative immunofluorescence images distinguishing fNRBCs (DAPI^+^/CD71^+^/CD45^−^) from leukocytes (DAPI^+^/CD45^+^/CD71^−^). (b) Scatter plot comparing fNRBC counts retrieved from peripheral blood of pregnant women *versus* non-pregnant controls. Statistical significance was determined using the Mann–Whitney *U* test (*p* < 0.001). (c) FISH analysis confirming the fetal origin of isolated cells using Y-chromosome specific probes (green signals).

Fig. 4a presents representative fluorescence microscopy images, demonstrating clear phenotypic discrimination between target fNRBCs and background WBCs. Quantitative analysis (Fig. 4b) revealed a highly significant difference in cell recovery between the two groups (Mann–Whitney *U* test, *p* < 0.001). Non-pregnant controls exhibited a negligible background count ranging from 0 to 1.5 cells per mL. This minimal background of DAPI^+^/CD71^+^/CD45^−^ cells likely originates from endogenous nucleated red blood cells (NRBCs) occasionally released into the peripheral circulation under mild physiological stress, or from trace non-specific antibody binding.

In sharp contrast, candidate fNRBCs were successfully detected in 100% of the maternal samples (*n* = 20). The isolated yields ranged from 10 to 83 cells per mL (detailed counts in Table S1). These results confirm that fNRBCs are consistently retrievable from maternal circulation throughout the second and third trimesters, with abundance fluctuations likely reflecting physiological variations in fNRBC trafficking during pregnancy.

### Definitive confirmation of fetal origin *via* FISH analysis

To provide conclusive evidence regarding the fetal identity of the isolated cells, we performed Fluorescence *In Situ* Hybridization (FISH) analysis. Compared to traditional morphological identification, FISH offers superior sensitivity and specificity for genetic verification.^15^ The assay was applied to cells isolated from two distinct sources: (1) umbilical cord blood from a male fetus (serving as a positive control), and (2) peripheral blood from pregnant women carrying confirmed male fetuses.

First, to validate the specificity of the probe protocol, we analyzed the positive control samples. As shown in Fig. S4, fNRBCs isolated from male cord blood exhibited distinct green fluorescence signals corresponding to the Y-chromosome centromere (CEP Y), confirming the efficacy of the hybridization. Subsequently, we extended this analysis to the clinical maternal blood samples. As definitively illustrated in Fig. 4c, a subpopulation of the isolated cells displayed clear Y-chromosome signals (Y^+^/DAPI^+^), unequivocally identifying them as male fetal cells. In sharp contrast, the surrounding background cells (maternal leukocytes) exhibited only DAPI nuclear staining (Y^−^/DAPI^+^), confirming the absence of the Y chromosome. Collectively, these FISH results provide compelling evidence that the cells retrieved by our cascaded microfluidic platform are indeed of fetal origin. This successful genotypic validation underscores the high reliability and specificity of our approach, highlighting its potential for non-invasive prenatal diagnosis of sex-linked genetic disorders and other chromosomal abnormalities. While Y-chromosome FISH provided definitive proof of fetal origin in this study, this validation method is inherently restricted to pregnancies with male fetuses, warranting the development of gender-independent genetic verification strategies for universal clinical applications.

## Conclusions

In summary, we have developed and validated an integrated two-step microfluidic platform for the highly efficient and specific isolation of fetal nucleated red blood cells (fNRBCs) from maternal blood. By synergistically combining label-free DLD enrichment with immunomagnetic negative purification, this cascaded system effectively overcomes the challenges posed by the extreme rarity of target cells and the complex maternal background. The platform demonstrated exceptional performance, achieving consistently high recovery efficiencies (>90%) in K562 spike-in experiments. Crucially, its clinical utility was robustly verified through the successful isolation and definitive identification of fNRBCs from 100% of tested maternal blood samples (*n* = 20), with fetal origin unequivocally confirmed *via* immunofluorescence phenotyping (DAPI^+^/CD71^+^/CD45^−^) and Y-chromosome FISH analysis.

This technology represents a pivotal advancement in cell-based non-invasive prenatal diagnosis (NIPD). By enabling the retrieval of intact, viable, and high-purity fetal cells, it circumvents the inherent limitations of cell-free fetal DNA (cffDNA) analysis—such as DNA fragmentation and maternal background interference. Consequently, our approach opens a promising avenue for comprehensive prenatal genetic testing, extending capabilities beyond aneuploidy screening to include the detection of micro-deletions, duplications, and monogenic disorders.

Despite the significant advantages in throughput and recovery, several limitations of the current platform should be discussed. First, the present workflow is semi-automated, requiring a manual off-chip magnetic bead incubation step between the two microfluidic stages; future iterations will incorporate on-chip active mixing modules to achieve true automation. Second, while a >99% leukocyte depletion is achieved, the absolute purity remains insufficient for direct bulk whole-genome sequencing (WGS) due to the persistent maternal cell background. Therefore, our platform serves as an ideal primary enrichment module that can be seamlessly coupled with high-efficiency downstream single-cell picking technologies (*e.g.*, our previously reported microfluidic single-cell isolation devices) to retrieve single fNRBCs for high-quality genomic analysis.^26^ Lastly, our genotypic validation *via* Y-FISH is currently restricted to pregnancies with male fetuses. To bypass this gender dependency, ongoing efforts focus on implementing gender-independent molecular diagnostics—specifically short tandem repeat (STR) analysis and fetal-specific epigenetic methylation profiling—to achieve universal clinical screening capabilities.

## Author contributions

Y. C. and Z. L. conceived the study concepts and supervised the project. H. F. and Y. H. designed and fabricated the microfluidic devices. Y. H., J. Z., S. G. and W. S. performed the experiments and analyzed the data. H. F. and F. S. performed the simulations. H. L., K. M. and L. X. contributed to the data analysis. H. F., B. H., and Y. H. drafted the original manuscript. Y. C. and Z. L. provided critical revisions and final approval. All authors discussed the results and contributed to the final manuscript.

## Conflicts of interest

The authors declare no competing interest.

## Supplementary Material

RA-OLF-D6RA02995G-s001

## Data Availability

The data underlying the presented results are available within the article and its supplementary information (SI). Supplementary information: schematic of the DLD separation principle (Fig. S1), COMSOL simulation of the magnetic field distribution (Fig. S2), live/dead staining fluorescence images of recovered cells (Fig. S3), FISH analysis images of isolated fNRBCs (Fig. S4), a summary table containing detailed counts and gestational ages of clinical blood samples (Table S1); and theoretical calculations of hydrodynamic and magnetic forces at the single-cell level (Note 1). See DOI: https://doi.org/10.1039/d6ra02995g.
