# Supplementary material for: High-efficiency isolation of fetal nucleated red blood cells for non-invasive prenatal diagnosis via a cascaded microfluidic platform
Source: RSC Adv. 2026 Jul 3;16(35):35546–54. doi: 10.1039/d6ra02995g (PMC13329752; doi:10.1039/d6ra02995g)
Supplement: RA-016-D6RA02995G-s001 [file RA-016-D6RA02995G-s001.pdf]

Supplementary Information (SI) for RSC Advances.
This journal is © The Royal Society of Chemistry 2026

High-Efficiency Isolation of Fetal Nucleated Red Blood Cells for Non-Invasive Prenatal Diagnosis via a Cascaded Microfluidic Platform

Hongtao Feng*†[a], Yuqing Huang†[a], Weiliang Shu[a], Fengshan Shen[a], Bin Huang[a], Jiaxin Zhang[a], Shunchan Gao[a], Hui Liang[c], Likuan Xiong[c], Kaidong Ma[d], Zongbin Liu*[b], and Yan Chen*[ae]

[a] *Shenzhen Institutes of Advanced Technology, Chinese Academy of Sciences, Shenzhen, 518055, China*

[b] *Shenzhen Zigzag Biotechnology Co., Ltd., Shenzhen, 518107, China*

[c] *Shenzhen Baoan Women's and Children's Hospital, Shenzhen, 518102, China*

[d] *Center of Obstetrics and Gynecology, Peking University Shenzhen Hospital, Shenzhen, 518036, China*

[e] *Shenzhen Raymind Biotechnology Co., Ltd., Shenzhen, 518129, China*

* *Corresponding author:*

Hongtao Feng (ht.feng@siat.ac.cn);

Zongbin Liu (zongbin.liu@zigbio.com);

Yan Chen (yan.chen@siat.ac.cn).

† *These authors contributed equally to this work.*

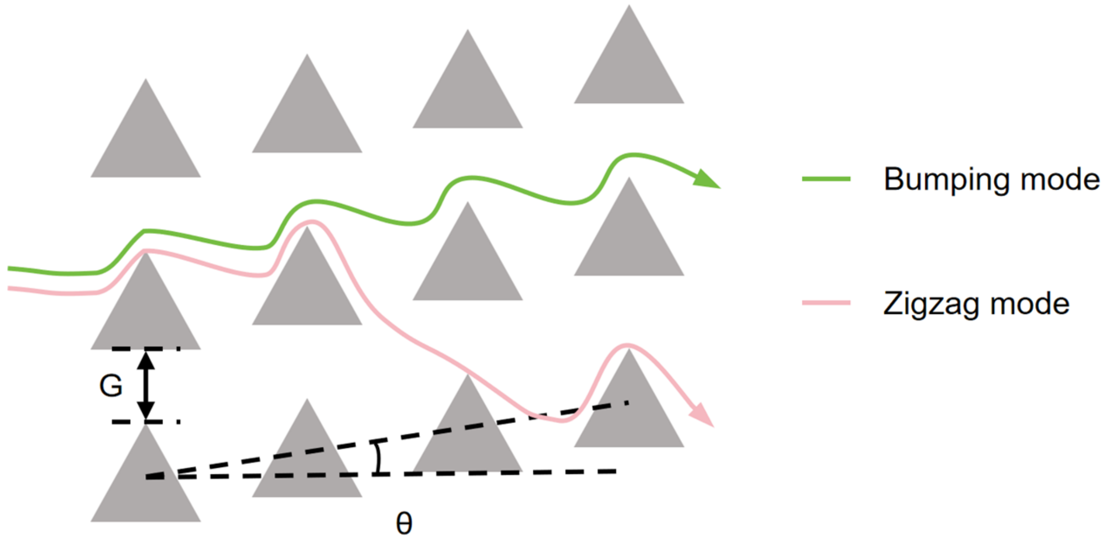

Fig. S1 Schematic illustration of the deterministic lateral displacement (DLD) separation principle. The key structural parameters, gap distance (G) and tilt angle (θ), determine the particle trajectories: smaller particles follow the fluid flow in a "zigzag" mode (pink line), while larger particles are laterally deflected in a "bumping" mode (green line).

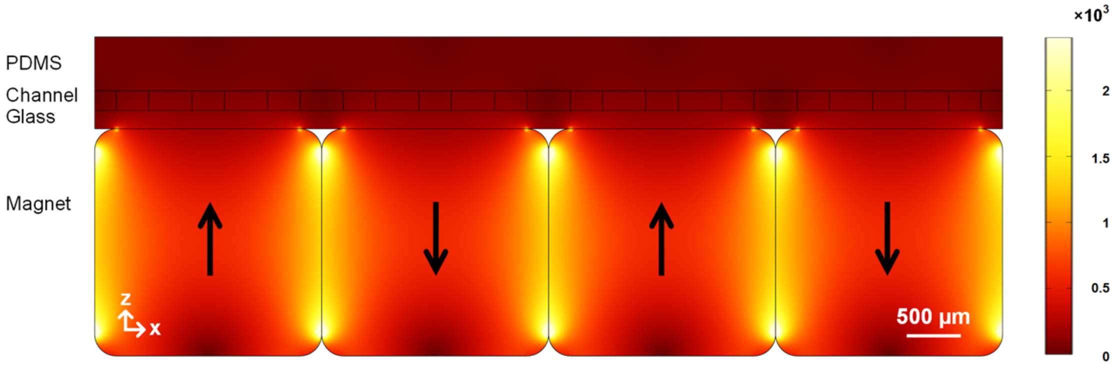

Fig. S2 COMSOL simulation of a magnetic field distribution within a microfluidic chip. (unit: mT).

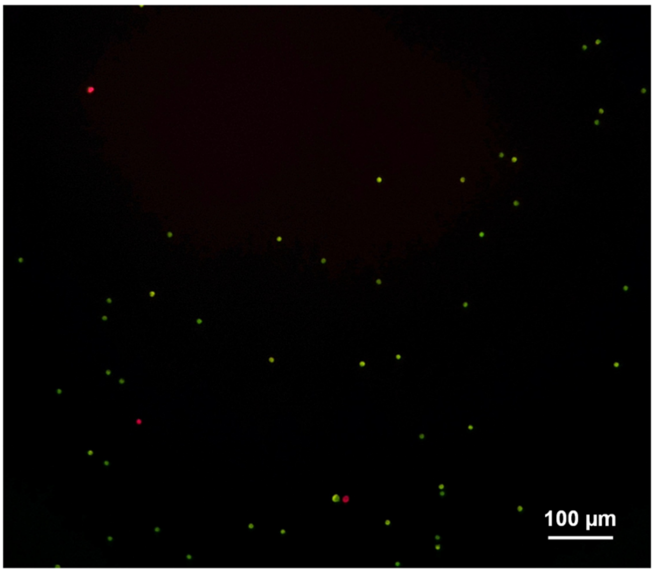

Fig. S3 Live/dead staining of recovered target cells after cascaded microfluidic processing. Fluorescence images show AO (live cells, yellow-green) and PI (dead cells, red) staining. Scale bar: 100 μm.

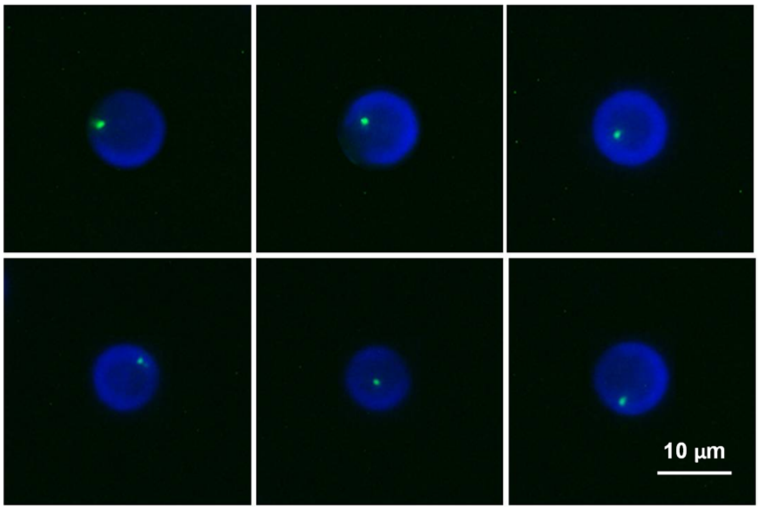

Fig. S4 Fluorescence in situ hybridization (FISH) analysis of fNRBCs isolated from male umbilical cord blood.

Table S1 Count of fNRBCs in pregnant and non-pregnant blood samples.

Sample ID	Volume (mL)	Age	Gestational Age (week^{+day})	fNRBC Count	fNRBCs/mL
			Pregnant Group		
1	5.0	33	26^{+2}	157	31.4
2	5.0	30	18^{+4}	132	26.4
3	2.0	33	14^{+3}	100	50
4	2.0	27	17^{+5}	78	39
5	2.0	29	19^{+4}	63	31.5
6	2.0	33	17^{+4}	42	21
7	2.0	31	17^{+1}	45	22.5
8	2.0	24	15^{+3}	89	44.5
9	2.0	27	17^{+1}	110	55
10	2.0	31	17^{+6}	105	52.5
11	2.0	32	17^{+4}	79	39.5
12	2.0	26	16^{+4}	65	32.5
13	2.0	23	17^{+0}	54	27
14	2.0	31	16^{+5}	76	38
15	2.0	37	18^{+3}	42	21
16	2.0	27	14^{+1}	36	18
17	2.0	32	17^{+5}	20	10
18	2.0	32	17^{+1}	26	13
19	2.0	32	21^{+2}	60	30
20	2.0	34	37^{+2}	166	83
			Control Group		
1	2.0	23	N/A	0	0
2	2.0	30	N/A	3	1.5
3	2.0	34	N/A	0	0
4	2.0	28	N/A	3	1.5
5	2.0	31	N/A	2	1

Supplementary Note 1: Theoretical Calculation of Force at the Single-Cell Level

To theoretically validate the capture efficiency of the Fe-doped magnetic microfluidic chip, we established a quantitative force balance model between the hydrodynamic drag force (F_{drag}) and the magnetic retention force (F_{mag}).

The hydrodynamic drag force acting on a spherical cell in a laminar flow field can be estimated using Stokes' law:

$$F_{drag} = 6\pi\eta R_c v_{rel}$$

where η is the dynamic viscosity of the medium, R_c is the cell radius (estimated at 6 μm), and v_{rel} is the fluid velocity around the cell (1 mm/s in simulation). The calculated F_{drag} exerted on a single leukocyte is approximately 113.1 pN.

Concurrently, the magnetic force acting on a single magnetic bead is given by:

$$F_{mag\_single} = \frac{V_b \Delta\chi}{\mu_0}(B \cdot \nabla)B$$

where V_b is the volume of a single bead (200 nm in diameter), $\Delta\chi$ is the magnetic susceptibility difference (empirically ~5, reported in Ref [1]), μ_0 is the vacuum permeability, and $(B \cdot \nabla)B$ represents the local magnetic field gradient term extracted from the COMSOL simulation. The individual magnetic retention force (F_{mag_single}) generated by the Fe-doped pillars is estimated at 2.89 pN.

Supplementary References:

[1] S. Bhuvanendran Nair Gourikutty, C.-P. Chang and P. D. Puiu, *J. Chromatogr. B*, 2016, **1011**, 77-88.
